# Multifactorial Hyponatremia: Don’t Miss Ruling Out Malignancy

**DOI:** 10.7759/cureus.35657

**Published:** 2023-03-01

**Authors:** Darby L Keirns, Alyssa Krautbauer, Diana Zamora, Mark M MacElwee

**Affiliations:** 1 Internal Medicine, Creighton University School of Medicine, Phoenix, USA; 2 Internal Medicine, Midwestern University Arizona College of Osteopathic Medicine, Glendale, USA; 3 Internal Medicine, Valleywise Health Medical Center, Phoenix, USA

**Keywords:** general internal medicine, multifactoral hyponatremia, polydipsia, differential diagnosis of hyponatremia, lung cancer, euvolemic hyponatremia

## Abstract

A 62-year-old male with a history of chronic obstructive pulmonary disease* *(COPD), schizoaffective disorder treated with Zoloft, type 2 diabetes mellitus, and tobacco use presented with an acute on chronic hyponatremia of 120 mEq/L. He presented with only a mild headache and endorsed recently increasing his free water intake due to a cough. Labs and physical exam findings suggested a true, euvolemic hyponatremia. Polydipsia and Zoloft-induced syndrome of inappropriate antidiuretic hormone (SIADH) were determined to be likely contributors to his hyponatremia. However, given his tobacco use, further workup was done to rule out malignancy causing hyponatremia. Chest CT did ultimately suggest malignancy and further workup was recommended. With the hyponatremia treated, the patient was discharged with recommended outpatient workup. This case serves as a reminder to consider that hyponatremia may be multifactorial and even if there is a likely cause identified, malignancy should be ruled out in patients with risk factors.

## Introduction

Hyponatremia, a condition characterized by low sodium levels (<135 mEq/L), is a common occurrence with a vast differential ranging from benign causes to malignancy. Hyponatremia can be characterized by acuity (acute, chronic) and severity (mild, moderate, severe) with widely ranging symptoms from nausea or headache to altered mental status and seizures [1-3]. Hyponatremia is often treated to prevent more serious manifestations, but it should also be evaluated to identify the underlying cause. Given the vast differential of hyponatremia, certain patients may have multiple underlying contributors to their presenting hyponatremia. The importance of addressing hyponatremia is also particularly underscored by previous observations that hyponatremia can precede clinical signs and symptoms of an underlying malignancy [1,4]. In the diagnostic evaluation of a patient presenting with hyponatremia, it is important to consider the possibility of a multifactorial etiology, including malignancy.

## Case presentation

**Figure 1:**
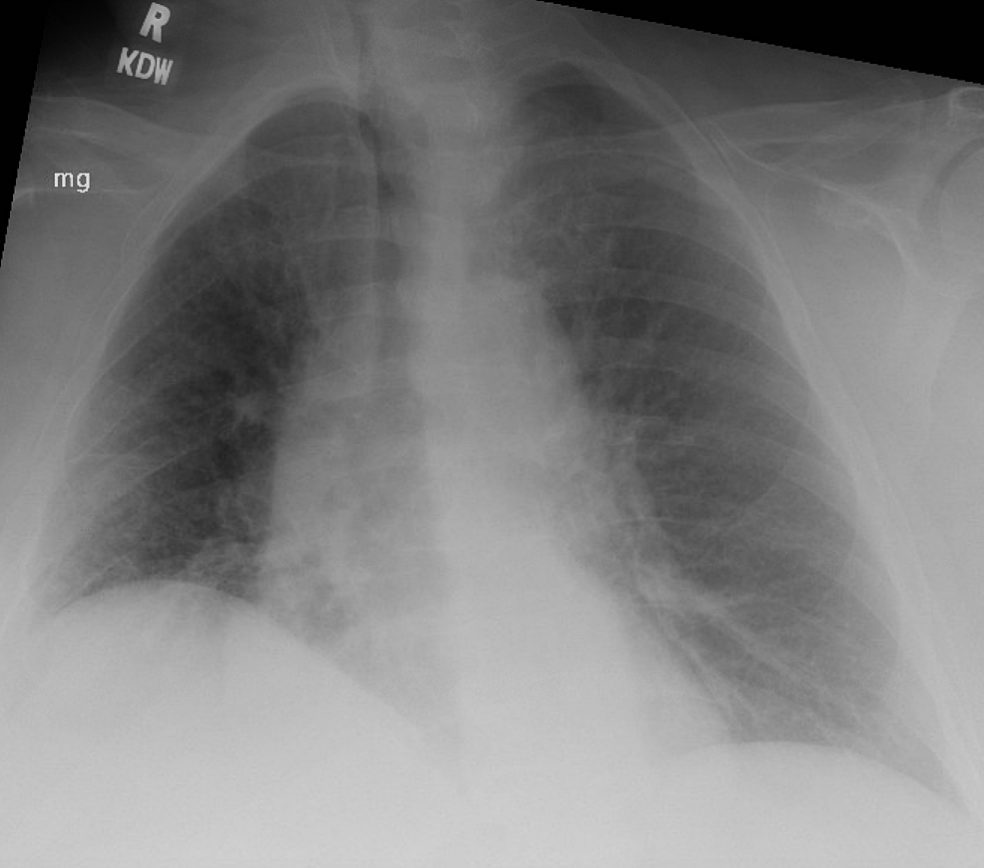
Initial chest X-ray The chest X-ray reveals interstitial opacities predominantly of the lower lung, suggestive of pulmonary edema. No obvious pulmonary masses are seen.

A 62-year-old male with a history of COPD on oxygen, schizoaffective disorder treated with Zoloft, and type 2 diabetes mellitus presented from an assisted living facility after labs revealed hyponatremia (120 mEq/L). Besides a mild headache, the patient was asymptomatic for hyponatremia, denying nausea, vomiting, weakness, or seizures. On review of symptoms, he did endorse shortness of breath, wheezing, and constipation. Recently, he increased his water intake to three to four 64 oz bottles per day for a mild cough. He also had a long history of smoking cigarettes and currently smoked tobacco from a pipe. During admission, he was hypertensive and hypoxic. On physical exam, he had diffuse wheezing and a distended abdomen. He appeared euvolemic with moist mucous membranes, capillary refill less than 2 seconds, with no jugular venous distension or edema. Initial labs were significant for hyponatremia (123 mEq/L; normal range 135-145 mEq/L), low serum osmolality (266 mOsmol/kg; normal range 275- 295 mOsmol/kg), hyperglycemia (171 mg/dL; normal range <140 mg/dL), low urine specific gravity (1.003; normal range 1.005-1.030), urine osmolality of 150 mOsm/kg H_2_O, and urine sodium of 24 mEq/L. A previous chart review revealed the patient had chronically low sodium around 129 mEq/L which had been documented at various intervals over the previous three years. A chest X-ray ordered in the emergency department demonstrated lower lung interstitial opacities and possibly pulmonary edema (Figure 1).

**Figure 2:**
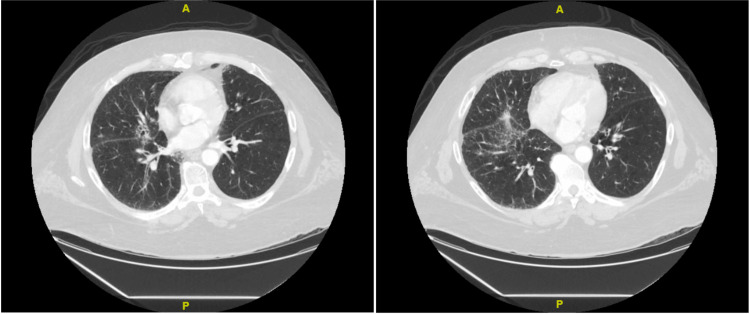
Chest CT The chest CT is suggestive of a diffuse and invasive type of adenocarcinoma with lymphangitic carcinomatosis.

Given the lab and exam findings, this patient was determined to have a true, euvolemic hyponatremia with a differential including polydipsia, syndrome of inappropriate antidiuretic hormone secretion (SIADH), malignancy, medications, hypothyroidism, or glucocorticoid deficiency, among others. Further workup including thyroid-stimulating hormone (TSH), cortisol level, and urine drug screen (UDS) was negative. Given the patient’s increased free water intake, polydipsia was a likely contributor and water restriction was begun. Additionally, Zoloft was discontinued as a potential cause of drug-induced SIADH. Although polydipsia and SIADH were reasonable explanations, malignancy was not ruled out. Given his smoking history, our team felt it important to further investigate with chest CT, the results of which suggested a diffuse and invasive type of adenocarcinoma with lymphangitic carcinomatosis (Figure 2). 

Pulmonology was consulted and bronchoscopy was recommended, which the patient did not wish to complete inpatient. Ultimately, with fluid restriction and salt tablets, his sodium returned to his baseline of 129 mEq/L and he was started on Abilify to replace Zoloft. He was discharged with arrangements for outpatient care for his newly discovered malignancy.

## Discussion

This case illustrates the importance of considering a broad differential and the possibility of multiple etiologies contributing to hyponatremia. Concluding that this patient’s hyponatremia was from medication and polydipsia solely would have stopped further workup and resulted in a missed diagnosis of cancer, even if it was non-contributory to the presenting hyponatremia.

Differential diagnosis

The differential diagnosis of hyponatremia is vast and often narrowed down based on plasma osmolality, volume status, urine sodium, and urine osmolality [5]. This patient was determined to have a true, euvolemic hyponatremia which most commonly includes a differential of polydipsia, SIADH, hypothyroidism, drug- or medication-induced SIADH, and adrenal insufficiency [6]. Given the patient’s history of increased free water intake and Zoloft usage, among normal TSH, cortisol, and UDS, it was concluded that polydipsia and Zoloft-induced SIADH were likely contributors. The urine osmolality of 150 mOsm/kg H_2_O further suggested SIADH, but given the moderate level and significant water intake, possible contribution by polydipsia in this multifactorial picture was still considered. Furthermore, recognizing this patient’s tobacco use, malignancy-induced SIADH was still on the differential. Further imaging suggested malignancy but without biopsy, there is no definitive conclusion or diagnosis of the type of cancer for this patient, although imaging suggested an invasive type of adenocarcinoma with lymphangitic carcinomatosis. In lung malignancy, hyponatremia is often recognized as a paraneoplastic SIADH in small cell lung cancer (SCLC). However, a growing body of research is shedding light on the occurrence and prognostic value of hyponatremia in non-small cell lung cancer (NSCLC) as well [7]. Ultimately, there is no definitive say whether this patient’s lung malignancy was a contributor to his hyponatremia, but proper workup of the hyponatremia allowed for it to be discovered.

Patient outcome and follow-up

The hyponatremia was treated with fluid restriction, salt tablets, and discontinuation of Zoloft. No treatment for lung cancer was initiated during the hospital stay as further workup was necessary. The patient declined further inpatient workup for lung cancer and wished to proceed as an outpatient, although he did not return for follow-up according to our records. Therefore, this patient’s long-term outcome is unknown.

Limitations

This case report is limited by the inability to determine definitively which factors were contributing or not, given the clinical picture and inability to isolate potential causes when treatment of water restriction and Zoloft discontinuation was provided simultaneously. It is further limited by a lack of definitive pathological diagnosis of lung cancer and absent patient follow-up.

## Conclusions

When evaluating hyponatremia, it is important to carefully assess all possible explanations and not limit further consideration by finding one fitting cause. Further, if there is a risk for malignancy, such as smoking history, it is wise to consider further investigation.
